# Model-Based Position and Reflectivity Estimation of Fiber Bragg Grating Sensor Arrays

**DOI:** 10.3390/s18072268

**Published:** 2018-07-13

**Authors:** Stefan Werzinger, Darko Zibar, Max Köppel, Bernhard Schmauss

**Affiliations:** 1 Institute of Microwaves and Photonics, Friedrich-Alexander University Erlangen Nürnberg, Cauerstr. 9, 91058 Erlangen, Germany; max.koeppel@fau.de (M.K.); bernhard.schmauss@fau.de (B.S.); 2 DTU Fotonik, Technical University of Denmark, Kgs. DK-2800 Lyngby, Denmark; dazi@fotonik.dtu.dk; 3 Graduate School of Advanced Optical Studies (SAOT), Friedrich-Alexander University Erlangen-Nürnberg, 91052 Erlangen, Germany; 4 Max-Planck Institute for the Science of Light, Staudtstr. 2, 91058 Erlangen, Germany

**Keywords:** optical fiber sensing, fiber Bragg gratings, signal processing, modeling, estimation of distribution algorithm, optical frequency domain reflectometry, temperature sensing

## Abstract

We propose an efficient model-based signal processing approach for optical fiber sensing with fiber Bragg grating (FBG) arrays. A position estimation based on an estimation of distribution algorithm (EDA) and a reflectivity estimation method using a parametric transfer matrix model (TMM) are outlined in detail. The estimation algorithms are evaluated with Monte Carlo simulations and measurement data from an incoherent optical frequency domain reflectometer (iOFDR). The model-based approach outperforms conventional Fourier transform processing, especially near the spatial resolution limit, saving electrical bandwidth and measurement time. The models provide great flexibility and can be easily expanded in complexity to meet different topologies and to include prior knowledge of the sensors. Systematic errors due to crosstalk between gratings caused by multiple reflections and spectral shadowing could be further considered with the TMM to improve the performance of large-scale FBG array sensor systems.

## 1. Introduction

Optical fiber sensors (OFS) are a powerful sensing technology in many industrial, medical or defense applications, offering unique properties. Especially, their immunity to electromagnetic interferences, chemical resistance, low signal attenuation and large multiplexing capacity makes them well suited for sensing under harsh conditions and on a large scale. In particular, reflectometer-based distributed OFS provide a huge amount of sensing points over many kilometers of range [1,2,3,4]. Conventional signal processing methods for such distributed OFS include optical pulse coding [5,6], Fourier transform [7,8,9] or wavelet transform processing [10,11]. In recent years, however, more and more advanced digital signal processing concepts, for example based on image processing [12] and machine learning [13], have been applied to further enhance the performance of distributed OFS.

Model-based signal processing [14,15] can be a very efficient technique in this context, as system states and sensor information can be estimated, using specifically tailored parametric models, also exploiting prior knowledge of the sensors. In this paper, we propose and demonstrate a novel model-based processing concept for quasi-distributed sensing with fiber Bragg grating (FBG) arrays, providing increased efficiency compared to conventional techniques, in particular Fourier transform processing [7]. The developed models provide great flexibility and could be easily expanded to meet different system configurations or could be used in connection with machine learning algorithms, for example.

FBGs are widely used OFS for temperature and strain measurements [16,17] or bio-sensing [18]. Their principle is based on the inscription of a longitudinal refractive index grating inside the core of a single mode fiber (SMF), using either a UV or femtosecond laser. If the wavelength of the incident light satisfies the Bragg condition, a strong narrow-band reflection from the grating can be measured. This so-called Bragg wavelength $\lambda_{B}$ is given by $\lambda_{B}=2n_{\mathrm{eff}}\Lambda$, where $n_{\mathrm{eff}}$ is the effective refractive index of the SMF and Λ the grating period [19]. Both of these parameters depend on temperature and strain and shift the Bragg wavelength accordingly. Once this relation is characterized for an FBG sensor, temperature and strain can be directly derived from a measurement of the Bragg center wavelength.

Usually, multiple FBGs are multiplexed, using wavelength division multiplexing (WDM) [20], where each FBG is designated a unique wavelength channel. However, more and more applications trend towards distributed sensing [3,4], where the position of a sensing element needs to be determined in addition to the actual readout or a large number of sensing points is required. In order to achieve this for FBGs, the excitation light can be modulated, so that the position of the gratings can be measured either from the time domain impulse response h(t) [21] or the corresponding frequency response H(f) [7]. This multiplexing is usually referred to as time division multiplexing (TDM), as the grating positions are determined from the propagation time of the light in the fiber. TDM significantly boosts the multiplexing capacity of the sensing system, as up to thousands of FBGs can be multiplexed by their position in each WDM channel [22,23].

Most TDM/WDM methods use the principle of optical time domain reflectometry (OTDR) [21,22,23], where a short pulse of high peak power, but relatively low energy is launched into the fiber. While this is quite straightforward to implement, a measurement of the equivalent frequency response H(f) with the technique of incoherent optical frequency domain reflectometry (iOFDR) provides some advantages, especially in regard to model-based processing. First of all, by operating in the frequency domain, system non-idealities and systematic errors can be accurately compensated by a calibration, resulting in an improved and more deterministic measurement of the sensor fiber response. Moreover, the multiplication property of the frequency domain allows one to concatenate the transfer functions of various fiber components by simple multiplications, instead of cumbersome convolutions, which would be required in the time domain. As a consequence, the conventional processing of transforming the measured iOFDR frequency domain response into the time domain, using an inverse Fourier transform (IFT) [7], would give this advantage away.

Additionally, Fourier transform processing is associated with some further issues and limitations. Most notably, the spatial two-point resolution must be chosen much higher than the distance between the FBGs, so that individual reflection peaks can be clearly separated from each other. Otherwise, crosstalk between nearby reflection peaks due to spectral leakage would appear. This requires an overly large electrical measurement bandwidth, increasing system costs [15]. While the spectral leakage effect could be well suppressed by windowing [24], this would further compromise the spatial resolution and introduce a processing loss.

In this paper, we will show that by replacing Fourier transform processing with model-based processing directly in the frequency domain, these issues can be avoided. We will also demonstrate that model-based processing outperforms Fourier transform processing, particularly near the spatial resolution limit, where it would be difficult to clearly separate nearby reflection peaks. With the model-based approach, a more efficient processing is possible and electrical bandwidth and measurement time can be saved. Furthermore, a powerful transfer matrix model (TMM) will be introduced, considering multiple reflections and spectral shadowing effects, which cause crosstalk errors in FBG arrays [21,25].

In Section 2, we first give a brief overview of the iOFDR measurement principle and describe the experimental setup. Then, the model-based estimation methods are explained in detail. We develop a parametric transfer matrix model (TMM) of the iOFDR frequency response of an FBG array, which is complemented by an estimation of distribution algorithm (EDA) for the position estimation. We evaluate the performance of our methods with Monte Carlo simulations and experimental data in Section 3. The results are further discussed in the context of previous work and large-scale FBG array sensing in Section 4. A final conclusion is given in Section 5.

## 2. Materials and Methods

### 2.1. Measurement Principle and Setup

The iOFDR technique can perform a spatially-resolved measurement of reflections and backscattering in an optical fiber for fault detection and diagnosis [26,27,28,29] and was also successfully applied to many sensing applications, including Raman distributed temperature sensing [9,30], dynamic strain sensing [31] and the quasi-distributed measurement of FBG arrays [7,30], which is also performed here. However, contrary to OTDR [32,33], which measures the time domain impulse response h(t), iOFDR uses radio frequency (RF) modulation signals to measure the corresponding frequency response H(f) in magnitude and phase. This frequency response is related to the time domain impulse response via a Fourier transform:

(1) $$H\left(f\right)=F\left\{h(t)\right\}.$$

In order to obtain the spatial reflectivity and backscatter profile $R\left(z\right)\propto \left|h(t)\right|$ of the fiber, an IFT can be applied to H(f), and the propagation time *t* is scaled to the distance *z* by the substitution $z=\frac{1}{2}v_{g}t$, where $v_{g}$ denotes the group velocity and the factor of 1/2 considers the forth and back propagation of the light. IOFDR is an incoherent technique that uses intensity modulation and direct detection of the light. This differs from coherent OFDR, where the wavelength of the light source is continuously tuned with high linearity and an interferometric detection is used [1,8]. For FBG multiplexing with iOFDR, the frequency response $H\left(f\right)=H\left(f_{k},\lambda_{n}\right)$ is measured at discrete modulation frequencies $f_{k}$ and wavelengths $\lambda_{n}$, which can be realized by using an electrical vector network analyzer (VNA) and a tunable light source, for example. In contrast to coherent OFDR however, the wavelength of the light source can be scanned step-wise with a much coarser sampling of several 10 picometers instead of highly linear continuous tuning.

**Figure 1:**
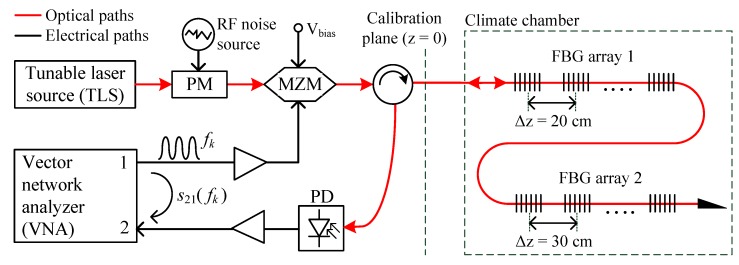
Wavelength scanning incoherent optical frequency domain reflectometer (iOFDR) measurement setup. MZM, Mach Zehnder modulator.

A wavelength scanning iOFDR setup for FBG array interrogation was introduced in [7] and is depicted in Figure 1. A tunable laser source (TLS, 81600B, Agilent Technologies, Santa Clara, CA, USA) performs a sequentially-stepped scanning of the wavelength. In order to fulfill the requirement of an incoherent light source, the coherence length $l_{c}$ of the TLS is artificially reduced, using a phase modulator (PM, LN65S-FC, Thorlabs, Newton, NJ, USA). By the phase modulation of the light source with broadband white noise, the line width of the laser is increased to about Δf≈ 6 GHz. This results in a coherence length of $l_{c}\approx$ 3.5 cm, effectively suppressing any interferences between spectrally-overlapping FBGs.

Due to the incoherent behavior of the light source, it is possible to use direct modulation and detection of the light intensity. A temperature-stabilized Mach Zehnder modulator (MZM, LN81S-FC, Thorlabs, Newton, NJ, USA) is biased in its quadrature operating point and used as an external amplitude modulator. The light received from the fiber is detected by a high-speed photodiode (PD, PQW20A-L, Albis Optoelectronics AG, Rueschlikon, Switzerland), converting the RF modulation signal back to the electrical domain.

The VNA (ZNB8, Rohde & Schwarz, Munich, Germany) generates a single frequency modulation signal. The modulation frequency $f_{k}$ is stepped in an equidistant grid (k=1,2,…,N) in steps of Δf. After the opto-electrical conversion, the received signal is filtered at an intermediate frequency (IF) and demodulated in magnitude and phase by the VNA, measuring the $s_{21}\left(f_{k}\right)$ parameter between ports 1 and 2. The IF filter bandwidth $B_{\mathrm{IF}}$ rejects most of the noise and any higher harmonics that might occur due to nonlinearities of the components (especially the MZM and driver amplifier). A narrower IF bandwidth improves the SNR, but also increases the sweep time of the VNA measurement.

One advantage of a frequency domain measurement setup is the accurate calibration capability of systematic errors and non-idealities of the electrical and electro-optical components. The calibration reference $s_{21,\mathrm{cal}}\left(f_{k}\right)$ is obtained by measuring the response from a fiber optic total reflector (R>97%) placed in the calibration plane as indicated in Figure 1. As the optical path isolates the receiving port 2 from the output port 1 in the backward direction, a simple transmission normalization of the iOFDR frequency response $H\left(f_{k}\right)=s_{21}\left(f_{k}\right)/s_{21,\mathrm{cal}}\left(f_{k}\right)$ already removes most of the systematic errors in the setup. This calibration normalizes the amplitude and phase of the system and also defines the spatial reference plane z=0.

### 2.2. Model-Based Estimation

The conventional processing of an iOFDR measurement uses an inverse discrete Fourier transform (IDFT) to obtain the spatially-resolved reflectivity profile R(z) from the measured frequency response $H(f_{k})$ at the TLS wavelength $\lambda_{n}$ [7]. However, there are some fundamental restrictions regarding the spatial resolution and range, well known from stepped-frequency radar [34]. The range is given by the maximum unambiguous length:

(2) $$L_{\max}=\frac{v_{g}}{2\Delta f},$$

depending on the frequency step size Δf, and the two-point spatial resolution is usually defined by:

(3) $$\Delta z_{\mathrm{res}}=\frac{v_{g}}{2B},$$

which is determined by the finite measurement bandwidth B=(N−1)Δf.

If we now assume a number of *M* FBG sensors at the same wavelength and in an equidistant spacing of ΔL, the spatial resolution needs to be much less than the spacings between the gratings $\Delta z_{\mathrm{res}}\ll \Delta L$, so that the individual reflection peaks can be clearly separated from each other. According to Equation (3), this requires an electrical measurement bandwidth $B\gg v_{g}/\left(2\Delta L\right)$. At the same time, the range condition $L_{\max}>M\Delta L$ must be fulfilled. As a result of Equations (2) and (3), the number of frequency points *N* that need to be measured will always significantly exceed the number of sensors N≫M, if conventional IDFT processing is applied. Considering that at a given measurement wavelength $\lambda_{n}$, only two parameters of a grating are actually required, in particular the position $z_{m}$ and reflectivity $R_{m}\left(\lambda_{n}\right)$, the frequency response $H(f_{k},\lambda_{n})$ exhibits a sparse character. This leads to the idea of a model-based processing, where the few required parameters are directly estimated from the frequency domain data using a sparse parametric model [15].

A model-based approach offers great flexibility and eases the restrictions of IDFT processing. We demonstrated that the bandwidth requirements can be reduced to a minimum of $B\approx v_{g}/\left(2\Delta L\right)$, saving system costs and also reducing the number of frequency points that need to be measured while offering a very good trade-off between measurement speed and precision [15]. In many cases, some prior knowledge of the sensor array will be available. This includes, for example, the number of FBG sensors *M* or their positions $z_{m}$, which may be known from manufacturing data or a prior measurement. A model-based approach takes advantage of this and allows one to include this prior knowledge, increasing the efficiency of the estimation for the remaining parameters. A modeling in the frequency domain is also attractive for mitigating crosstalk effects like spectral shadowing and multiple reflections [21,25] by transfer matrices, due to the convenient multiplication property of the frequency domain. This will be fully exploited in the following by a newly-developed transfer matrix model.

#### 2.2.1. Transfer Matrix Model

Transfer matrix models (TMM) are a powerful and flexible modeling approach to compose complex structures by concatenating several basic building blocks. The TMM method has been widely used to calculate the spectral response of FBGs, by discretizing the grating structure into many piecewise-uniform sections [35]. Based on coupled mode theory [36], this approach considers the interaction of the forward and backward propagating optical wave inside a grating. Here, we transfer the TMM idea to the interrogation of quasi-distributed FBG arrays with the iOFDR measurement technique. The main difference is that due to the incoherent nature of iOFDR, we derive transfer matrices in terms of the forward and backward modulation signal instead of the optical wave propagation. Consequently, a FBG will be modeled from a more macroscopic point of view, represented by a single transfer matrix. This is feasible, because the iOFDR spatial resolution is usually much coarser than the length of a grating, so that an FBG can be simply treated as a wavelength-dependent point reflector.

**Figure 2:**
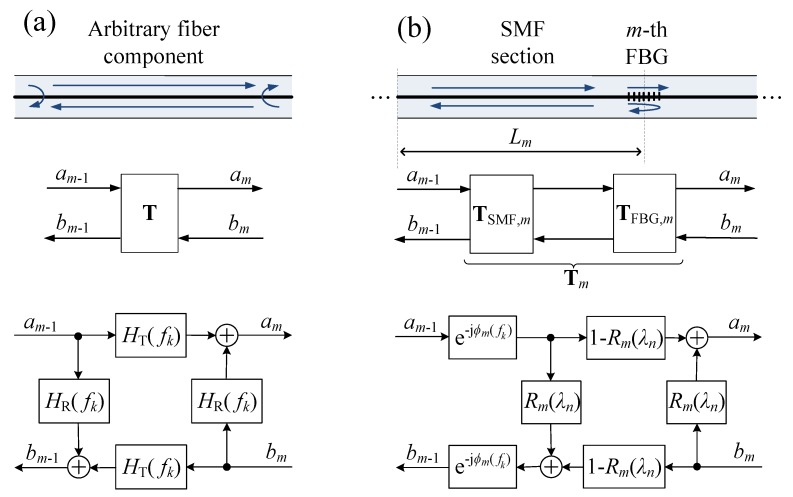
(**a**) Model representation of the transfer matrix of an arbitrary fiber component, decomposed in a transfer function in transmission and reflection $H_{T}\left(f_{k}\right)$ and $H_{R}\left(f_{k}\right)$, respectively. (**b**) Model representation of an FBG fiber span with a single mode fiber (SMF) section followed by a grating.

In general, any optical fiber section or component can be described by such a transfer matrix T in the iOFDR frequency domain, linking the (generally complex) forward propagating modulation signal $a_{m}$ and its backward propagating counterpart $b_{m}$, as depicted in Figure 2a. By multiplying several transfer matrices (m=1,2,…,M), the transmission and reflection of any configuration of components can be calculated, also covering multiple reflections, for example. The transfer matrix T of a component can be further decomposed in terms of its transfer functions $H_{T}\left(f_{k}\right)$ and $H_{R}\left(f_{k}\right)$ characterizing its transmission and reflection properties, respectively. This is very convenient, because for many components, $H_{T}\left(f_{k}\right)$ and $H_{R}\left(f_{k}\right)$ can be modeled or obtained from measurements. Assuming that T describes a linear system, the superposition principle applies, and the decomposition can be derived from the signal flowchart in Figure 2a, resulting in:

(4) $$\left(\begin{array}{c} a_{m}\\ b_{m} \end{array}\right)=\underset{T}{\underset{︸}{\left(\begin{array}{cc} T_{11} & T_{12}\\ T_{21} & T_{22} \end{array}\right)}}\cdot \left(\begin{array}{c} a_{m-1}\\ b_{m-1} \end{array}\right)=\frac{1}{H_{T}\left(f_{k}\right)}\left(\begin{array}{cc} H_{T}^{2}\left(f_{k}\right)-H_{R}^{2}\left(f_{k}\right) & H_{R}\left(f_{k}\right)\\ -H_{R}\left(f_{k}\right) & 1 \end{array}\right)\cdot \left(\begin{array}{c} a_{m-1}\\ b_{m-1} \end{array}\right).$$

We want to point out that the proposed frequency domain TMM approach is very flexible and can be easily extended in complexity. Various components can be concatenated in any order by simple multiplications of their transfer matrices. At this point, the multiplication property of the frequency domain turns out to be very favorable, compared to the time-domain, where more cumbersome convolutions would have to be calculated.

An FBG array with *M* gratings can be split into separate spans, each consisting of a fiber section with a physical length $L_{m}$, followed by a grating, as illustrated in Figure 2b. The transfer matrices of these components are derived under the following assumptions:

- All fiber-optic components are reciprocal and fulfill the requirements of linear and time-invariant systems.
- Typically, the length of a grating in an FBG array is in the order of some millimeters, and the peak reflectivities are well below 5% to minimize interferences by crosstalk and power depletion [25]. As the spatial resolution of the iOFDR measurement system is in the range of centimeters and the group delay of a weakly-reflecting grating is very small, the gratings are modeled as wavelength-dependent point reflectors with reflectivity $R_{m}\left(\lambda_{n}\right)$ in good approximation.
- Any losses of the gratings due to scattering or coupling to cladding modes are neglected, so that the transmitted and reflected power equals 100% of the incident light. This leads to a simplified FBG transfer matrix of:
  (5) $$T_{\mathrm{FBG},m}=\frac{1}{1-R_{m}\left(\lambda_{n}\right)}\left(\begin{array}{cc} 1-2R_{m}\left(\lambda_{n}\right) & R_{m}\left(\lambda_{n}\right)\\ -R_{m}\left(\lambda_{n}\right) & 1 \end{array}\right).$$
- For short fiber lengths, attenuation and dispersion effects are negligible, so that the optical fiber behaves like an allpass, introducing just a phase shift $\phi_{m}\left(f_{k}\right)$ due to the propagation delay. Furthermore, the weak Rayleigh backscattering in the fiber can usually be neglected, resulting in a diagonal transfer matrix for modeling a short SMF section:
  (6) $$T_{\mathrm{SMF},m}=\left(\begin{array}{cc} e^{-j\phi_{m}\left(f_{k}\right)} & 0\\ 0 & e^{j\phi_{m}\left(f_{k}\right)}. \end{array}\right)$$

The above assumptions are met in the demonstration experiment and most short and weakly reflecting FBG arrays, but are not ultimate constraints. Thanks to the flexibility of the transfer matrix approach, the above models could be further expanded in complexity to include effects like dispersion, attenuation, scattering, etc., if required.

The transfer matrix of an SMF fiber span followed by an FBG is graphically illustrated in Figure 2b and can be calculated by multiplying the individual transfer matrices (5) and (6), resulting in:

(7) $$\begin{array}{c} T_{\mathrm{Span},m}=T_{\mathrm{FBG},m}\cdot T_{\mathrm{SMF},m}=\left(\begin{array}{cc} \left[1-\rho_{m}\left(\lambda_{n}\right)\right]e^{-j\phi_{m}\left(f_{k}\right)} & \rho_{m}\left(\lambda_{n}\right)e^{j\phi_{m}\left(f_{k}\right)}\\ -\rho_{m}\left(\lambda_{n}\right)e^{-j\phi_{m}\left(f_{k}\right)} & \left[1+\rho_{m}\left(\lambda_{n}\right)\right]e^{j\phi_{m}\left(f_{k}\right)} \end{array}\right), \end{array}$$

with the abbreviations:

(8) $$\rho_{m}\left(\lambda_{n}\right)=\frac{R_{m}\left(\lambda_{n}\right)}{1-R_{m}\left(\lambda_{n}\right)},\;\;\mathrm{and}\;\;\phi_{m}\left(f_{k}\right)=\frac{2\pi f_{k}}{v_{g}}L_{m}.$$

In Equation (8), $R_{m}\left(\lambda_{n}\right)$ denotes the reflectivity profile of the *m*-th grating, $L_{m}$ is the physical length of its preceding fiber section and $f_{k}$ is the modulation frequency of the VNA. With the boundary condition $b_{M}\equiv 0$, the complex frequency response of a grating array with *M* spans is:

(9) $$H_{\mathrm{TMM}}\left(f_{k},\lambda_{n}\right)=\frac{b_{0}}{a_{0}}=-\frac{{\left(\prod_{m=M}^{1}T_{\mathrm{Span},m}\right)}_{21}}{{\left(\prod_{m=M}^{1}T_{\mathrm{Span},m}\right)}_{22}}.$$

Note that due to the definition of the matrix product:

(10) $$\prod_{m=a}^{b}T_{m}=T_{a}\cdot T_{a+1}\cdot \ldots \cdot T_{b-1}\cdot T_{b}=\left(\begin{array}{cc} {\left(\prod_{m=a}^{b}T_{m}\right)}_{11} & {\left(\prod_{m=a}^{b}T_{m}\right)}_{12}\\ {\left(\prod_{m=a}^{b}T_{m}\right)}_{21} & {\left(\prod_{m=a}^{b}T_{m}\right)}_{22} \end{array}\right),$$

the indices in (9) are numbered from high to low to give the correct multiplication order of the matrices.

#### 2.2.2. Position Estimation Method

As mentioned before, a FBG is basically described by two parameters, namely its position $z_{m}$ and reflectivity $R_{m}\left(\lambda_{n}\right)$ at the particular measurement wavelength $\lambda_{n}$. From the equations of the TMM (7) and (8), it can be seen that the span lengths $L_{m}$ and therefore the positions of the gratings determine the phase of the $e^{\pm j\Phi_{m}\left(f_{k}\right)}$ terms, while the reflectivities determine the amplitudes of these phasors. This makes a joint estimate of the positions and reflectivities quite difficult at wavelengths far from a Bragg resonance, where the reflectivity is very weak or almost zero. In order to address this, we propose a two-step procedure, where the positions are estimated independently of the reflectivities in a first step. After that, these estimated positions $z_{m}^{*}$ are used as static parameters in the TMM, so that only the reflectivities remain to be estimated.

In the following, we describe an estimation of distribution algorithm (EDA) [37,38] for estimating the positions of the Bragg sensors. EDAs are a class of evolutionary algorithms that perform well in many complex optimization problems and provide a certain amount of adaptability, which is particularly useful if the initial parameters are subject to uncertainty. In our case, an EDA-based procedure will allow us to specify the initial position guesses with increased robustness and also to compensate for position variations due to manufacturing tolerances.

Following the structure of a basic EDA, an initial population of candidate solutions is generated from an explicit probabilistic model, first. Then, the candidate solutions are evaluated by a fitness function, and the most promising solutions are selected, while the others are discarded. Analyzing the selected solutions, the probabilistic model is updated, and a new generation of candidate solutions is generated. This evolutionary update procedure is repeated for a certain number of iterations or until a certain quality of the solution is reached. Based on the EDA principle, we implemented a position estimation algorithm, which is outlined in Algorithm 1.

**Algorithm 1** Position estimation.
1. Sum the measured frequency response $H(f_{k},\lambda_{n})$ over all wavelengths $\lambda_{n}$:
(11)$$\bar{H}\left(f_{k}\right)=\sum_{n}H\left(f_{k},\lambda_{n}\right)$$2. Specify the initial grating positions $\mu_{m,0}$ and corresponding standard deviations $\sigma_{m,0}$.
**for** $j=0,1,\ldots ,N_{\mathrm{update}}$ **do**
    **for** $i=1,2,\ldots ,N_{\mathrm{population}}$ **do**        3. Draw *M* candidate positions $z_{m,i}^{*}$ from the probability densities $p_{m,j}\left(z\right)∼N\left(\mu_{m,j},\sigma_{m,j}^{2}\right)$.        4. Calculate the reflectivities ${\bar{R}}_{m,i}^{*}$ resulting from the solution of the least squares problem:
(12)$$\min_{{\bar{R}}_{m,i}^{*}}\sum_{k=1}^{N}{\left|\bar{H}\left(f_{k}\right)-\sum_{m=1}^{M}{\bar{R}}_{m,i}^{*}e^{-j\frac{4\pi f_{k}}{v_{g}}z_{m,i}^{*}}\right|}^{2},$$           using the closed form expression from Appendix A.        5. Calculate the mean squared error ${\mathrm{MSE}}_{i}$ between the estimated and measured frequency           response $\bar{H}\left(f_{k}\right)$.    **end for**    6. Calculate the *q*-th quantile of the mean squared errors ${\mathrm{MSE}}_{i}$ as a threshold.    7. Select only positions $z_{m,i}^{*}$ that resulted in an MSE below the threshold and discard all others.    8. Calculate the sample mean $\mu_{m,j+1}$ and variance $\sigma_{m,j+1}^{2}$ of the selected positions and       update the sampling distributions $p_{m,j+1}\left(z\right)∼N\left(\mu_{m,j+1},\sigma_{m,j+1}^{2}\right)$.
**end for**
9. Select the positions $z_{m,i}^{*}$ with the lowest MSE as the solution.


First, the estimation is made independently of the wavelength and individual reflectivities by summing the frequency response $H(f_{k},\lambda_{n})$ over all wavelengths. This ensures that every FBG is covered regardless of their actual Bragg wavelength and reduces the data to a one-dimensional vector $\bar{H}\left(f_{k}\right)$. By doing so, we neglect crosstalk effects like multiple reflections and spectral shadowing [21,25], yielding a simplified frequency response model as already used in [15], which only considers direct reflections:

(13) $$H\left(f_{k},\lambda_{n}\right)\approx \sum_{m=1}^{M}R_{m}\left(\lambda_{n}\right)e^{-j\frac{4\pi f_{k}}{v_{g}}z_{m}}.$$

This simplified model is linear in terms of the reflectivity parameters $R_{m}\left(\lambda_{n}\right)$, so (11) becomes:

(14) $$\bar{H}\left(f_{k}\right)\approx \sum_{m=1}^{M}\left[\sum_{n}R_{m}\left(\lambda_{n}\right)e^{-j\frac{4\pi f_{k}}{v_{g}}z_{m}}\right]=\sum_{m=1}^{M}{\bar{R}}_{m}e^{-j\frac{4\pi f_{k}}{v_{g}}z_{m}},$$

where ${\bar{R}}_{m}$ denotes arbitrary reflectivities that result from the wavelength summing and $z_{m}$ are the positions of the gratings relative to the calibration plane.

For the probabilistic model of the positions, we choose simple normal distributions with a mean value $\mu_{m,j}$ and a standard deviation $\sigma_{m,j}$, which are treated as stochastically independent. Usually, the nominal spacing ΔL and positions of the gratings are approximately known from manufacturing or an independent measurement, so we initially set $\mu_{m,0}$ to these nominal or measured values and allow a relatively coarse sampling of the positions at the beginning by setting $\sigma_{m,0}=\Delta L/2$.

A population of $i=1,2,\ldots ,N_{\mathrm{population}}$ proposal position vectors $z_{m,i}^{*}$ of length *M* is drawn from the distributions $p_{m,j}\left(z\right)$. Given these proposals, the simplified model (14) forms a linear equation system in terms of the reflectivities, which can be solved analytically in a least squares sense (see Appendix A), providing estimation values for ${\bar{R}}_{m,i}^{*}$. In order to quantify the fitness of the proposal positions, the mean squared error ${\mathrm{MSE}}_{i}$ between the measured $\bar{H}\left(f_{k}\right)$ and the estimated frequency response according to (14) is calculated. If the randomly drawn positions are close to the true positions, a low MSE will result.

**Figure 3:**
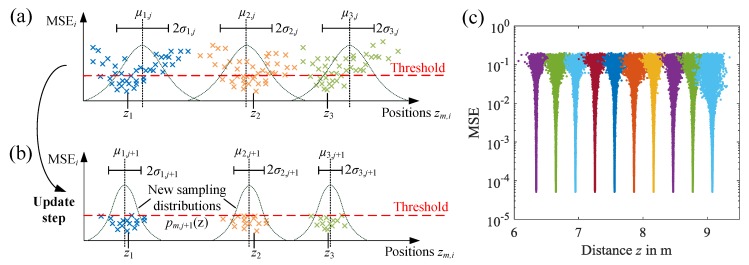
(**a**) Illustration of the estimation of distribution algorithm (EDA)-based position estimation algorithm, sampling the position parameters $z_{m,j}$ from initial probability distributions and calculating the global MSE as a fitness function. (**b**) Candidate positions resulting in MSE values above a threshold are discarded and the statistics of the selected positions are calculated to update the sampling distributions. (**c**) Example of the evolution of the MSE for the position estimation of the last 10 gratings in a measurement.

We perform $N_{\mathrm{update}}$ update steps of the sampling distributions, by analyzing the statistics of the most promising proposal positions $z_{m,i}^{*}$. These are selected by applying a threshold given by a certain quantile *q* of the ${\mathrm{MSE}}_{i}$ values. All position proposals that correspond to MSE values that exceed this threshold are discarded, and the sample means $\mu_{m,j+1}$ and variances $\sigma_{m,j+1}^{2}$ of the remaining position proposals are calculated. The sampling distributions for the next batch are then updated according to these sample means and variances. This selection and update step is also illustrated in Figure 3a,b. After several update steps, the sampling distributions will narrow and their centers approach the true positions.

**Figure 4:**
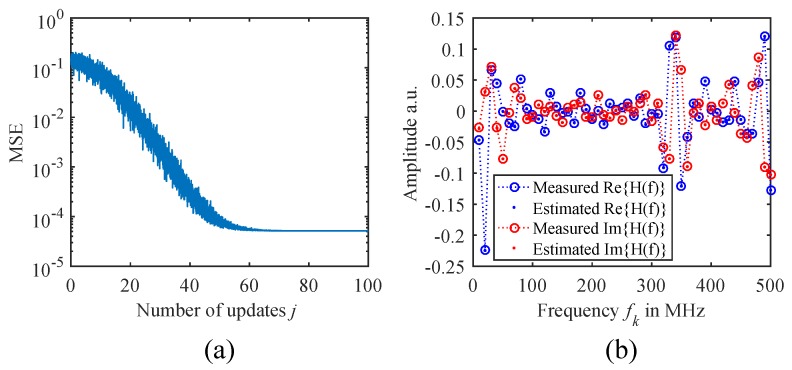
(**a**) Convergence of the MSE in the position estimation algorithm vs. the number of update steps. (**b**) Estimated real and imaginary parts of a measured frequency response $\bar{H}\left(f_{k}\right)$.

In our study, we chose the median q=0.5 as the MSE threshold and a population size of $N_{\mathrm{population}}=200$. This will always provide $qN_{\mathrm{population}}=100$ selected position sets, from which the statistics are calculated. Figure 3c shows an example of the MSE evolution for a measurement of an FBG array with ten gratings in a nominal spacing of ΔL=30cm after $N_{\mathrm{update}}=100$ update iterations. It can be seen that at the beginning, the samples are distributed over a broad range, yielding large MSEs. Due to the self-adjustment of the sampling distributions that the EDA provides, the algorithm is able to compensate position uncertainties to a certain extent, and after several update steps, the probability density functions narrow down and converge towards the minimum MSE solution. Figure 4a also shows that the MSE eventually converges if enough update steps are performed. In this example, the residual MSE is only limited by the amount of noise in the measurement data. The resulting fit of the frequency response $\bar{H}\left(f_{k}\right)$ is depicted in Figure 4b. It can be seen that the measured (circles) and fitted values (dots) of the real and imaginary parts match very well, demonstrating that the algorithm is working correctly.

#### 2.2.3. Reflectivity Estimation with TMM

After performing the position estimation, the actual reflectivities $R_{m}\left(\lambda_{n}\right)$ are estimated, using the TMM that is described in Section 2.2.1. As the positions have been determined before, the estimation problem reduces to the reflectivities only. In contrast to the position estimation, the reflectivities do not occur in the phase terms of the TMM equations, so their estimation works very well in a quite standard fashion, directly applying a least squares fit algorithm. Note however that the TMM equations are nonlinear in terms of the reflectivity parameters because many multiplications occur, so a nonlinear least squares solver is required for the estimation.

The TMM fit algorithm is implemented in MATLAB (R2016a), using the built-in lsqcurvefit() function with the trust-region-reflective solver. This solver allows the specification of the boundary constraints $0\leq R_{m}\left(\lambda_{n}\right)\leq 1$ for the reflectivities and can also be used for complex data if the imaginary and real parts are split. The initials for the reflectivities are set to zeros, so the fit algorithm only needs to know the number of gratings *M* and the estimated positions $z_{m}^{*}$ at the beginning. Moreover, the computation can be greatly accelerated by passing the elements of the Jacobian matrix to the solver, consisting of the partial derivatives $\partial H_{\mathrm{TMM}}\left(f_{k},\lambda_{n}\right)/\partial R_{m}\left(\lambda_{n}\right)$, which can be explicitly calculated as given in Appendix B. Finally, the reflectivities $R_{m}\left(\lambda_{n}\right)$ of all gratings are estimated for each wavelength $\lambda_{n}$ separately, resulting in the reflectivity profiles $R_{m}\left(\lambda_{n}\right)$ from which the Bragg center wavelengths can be extracted.

### 2.3. Algorithm Evaluation with Monte Carlo Simulations

In this section, we evaluate the proposed position estimation algorithm and the TMM fit algorithm with Monte Carlo simulations. This has the advantage that the true positions, reflectivities and Bragg wavelengths of the FBGs are exactly known, so stochastic and systematic errors of the estimation can be analyzed. The Monte Carlo data are generated by using the TMM for two FBG arrays, which are very similar to the constellation in the measurement setup in Figure 1. The Bragg wavelengths $\lambda_{B,m}$, spectral full width half maximum (FWHM) bandwidth $\Delta \lambda_{\mathrm{FWHM}}$, peak reflectivities $R_{B,m}$ at the Bragg wavelengths and the grating positions $z_{m}$ are specified according to Table 1. Additionally, some random variations of the nominal values are introduced to account for manufacturing tolerances and variations of the Bragg wavelengths due to temperature or strain in the simulations.

For weak gratings (R<0.2), the reflection coefficient can be calculated by a Fourier transform of the index modulation Δn(z) [19].According to this, the power reflection spectrum of a weak and uniform FBG can be well described by a ${\mathrm{sinc}}^{2}$-function:

(15) $$R_{m}\left(\lambda_{n}\right)=R_{B,m}\cdot {\mathrm{sinc}}^{2}\left(0.886\frac{\lambda_{n}-\lambda_{B,m}}{\Delta \lambda_{\mathrm{FWHM}}}\right).$$

Given the grating positions $z_{m}$ and the other parameters from Table 1, the frequency responses $H(f_{k},\lambda_{n})$ are simulated with the TMM.

Complex Gaussian noise with a root mean square (RMS) value of $\sigma_{n}=1.5\times 10^{-5}$ is added to the simulation data. The amount of noise was chosen so that the minimum MSE in the position estimation algorithm approximately matches the one observed with the experimental data in Figure 4a. In total, $N_{\mathrm{sim}}=1000$ frequency responses with slightly randomized parameters are generated. Just 50 modulation frequencies $f_{k}$ are evaluated, ranging from 10MHz–500MHz in steps of Δf=10MHz. According to (2) and (3), this would correspond to a range of $L_{\max}$ = 10.4 m and a two-point spatial resolution of $\Delta z_{\mathrm{res}}$ = 20.7 cm. With these parameters, the spatial resolution is already at the very limit for the FBGs in distances of 20 cm.

#### 2.3.1. Evaluation of Position Estimation

**Figure 5:**
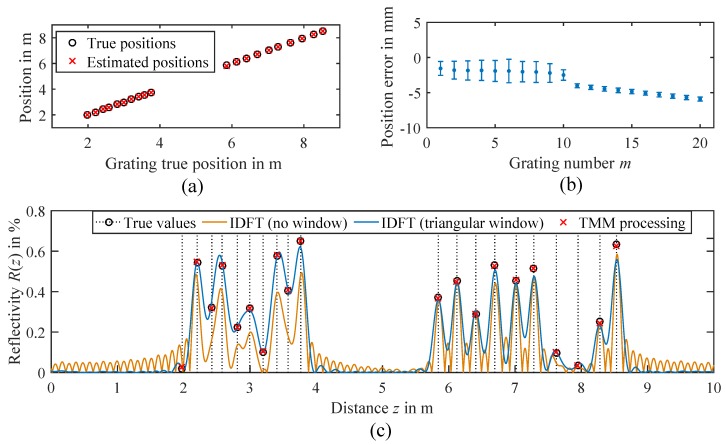
Position estimation errors in the Monte Carlo simulation. All examples are shown at a wavelength of $\lambda_{n}=1550\;\mathrm{nm}$. (**a**) Comparison of the true and estimated positions for one estimation. (**b**) Mean and standard deviation of the position error of each individual grating from all simulation data. (**c**) Comparison of the proposed TMM processing with conventional inverse discrete Fourier transform (IDFT) processing and windowing.

First, the performance of the position estimation with the EDA-based algorithm 1 is examined. The nominal positions of the FBGs in spacings of 20 cm and 30 cm are used as the initial means $\mu_{m,0}$ of the sampling distributions $p_{m,0}\left(z\right)$. However, as we always added some random position offsets of a few centimeters in the simulation data, the algorithm never starts at the true positions and is forced to converge correctly. Figure 5a shows an example of the resulting estimated positions in comparison to the true positions, which are in very good agreement. In order to evaluate the position error in more detail, the mean and standard deviation of the errors from all simulation runs are calculated and plotted in Figure 5b. The position error for the first and second array shows an average standard deviation of 1.3 mm and 0.3 mm, respectively. We think that this difference is mainly caused by the bandwidth limitation to 500 MHz, which is already the spatial resolution limit of the first array with a 20cm grid. However, in terms of absolute accuracy, the second array shows larger offsets of up to −5mm. This may be a result of the simplification made in the position estimation Equation (13), where only direct reflections are considered. Nevertheless, the errors are still relatively small and within 2% of the nominal spacing ΔL=30cm, so that the simplification of the position estimation model appears to be reasonable.

#### 2.3.2. Evaluation of Reflectivity Estimation Using TMM

The estimated positions are then used as static parameters in the TMM, so only the reflectivities of the gratings remain to be estimated. Figure 5c shows an example of the estimated reflectivities at a wavelength of $\lambda_{n}=1550\;\mathrm{nm}$, which match the true values very well. For comparison, the reflectivity trace R(z) obtained with conventional IDFT processing and zero padding according to [7] is also plotted. One trace shows the IDFT result without any windowing. In this case, large side lobes overlap with the direct reflections, making a correct evaluation of the peaks quite unfeasible. This problem is widely known as the leakage effect in signal processing and can be reduced by the use of a window function [24].

In the second trace, a triangular window was applied before the IDFT. Though a triangle is a very simple window, it will already illustrate the most relevant aspects. The side lobes are now suppressed by a sufficient amount, but at the same time, the widths of the main lobes increase, decreasing the effective spatial resolution. This will reveal stronger reflections more prominently, but an identification of weaker reflections is still not possible in every case. This is especially true near the spatial resolution limit. While the identification of the reflection peaks in the second array with ΔL=30cm works quite well, it is very difficult for the first array with ΔL=20cm. For example, the ninth FBG at about 3.5 m is completely covered by the neighboring reflections, so it cannot be identified. Moreover, any windowing will introduce additional processing losses that decrease the signal to noise ratio (SNR). Using a triangular window for example results in a 50% loss [24], so that the windowed trace in Figure 5c was scaled by a factor of two to compensate for that.

In contrast to IDFT processing, the model-based approach performs much better, and the reflectivities and positions of the gratings are still correctly determined, even if the reflectivities are low. No trade offs due to windowing and the associated decrease in spatial resolution and processing losses have to be made. As a result, a much more efficient processing of the bandwidth limited measurement data is possible compared to conventional IDFT processing, especially near the spatial resolution limit.

#### 2.3.3. Evaluation of Bragg Wavelength Errors

**Figure 6:**
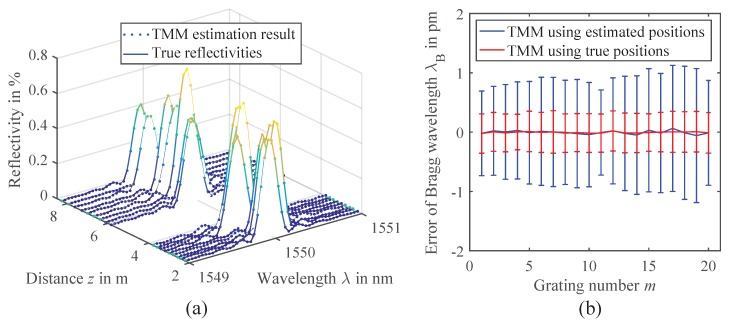
(**a**) Results of the position and TMM reflectivity estimation (dots) compared to the reference reflectivities (solid lines) from a single simulation run. (**b**) Mean and standard deviations of the Bragg wavelength errors determined from the simulation data. TMM estimation using the estimated positions is compared to the TMM estimation using the true grating positions as an input.

Most important for FBG sensing of course is the Bragg wavelength interrogation, which is analyzed next. Figure 6a shows the reflectivity estimation result for one constellation of the simulated FBG arrays. It can be seen that the reflectivity profiles can be reconstructed very well by the TMM algorithm, as the estimated reflectivities (dots) exactly match the specified true reflectivities (lines). In the next step, the Bragg wavelengths $\lambda_{B,m}$ are extracted from the estimated reflectivity profiles. A variety of methods exists for the Bragg peak detection [39]. For simplicity, we chose to fit a Gaussian function to the reflectivity profiles, which is a simple and common method, working well with the ${\mathrm{sinc}}^{2}$-shaped profiles (15) of weak and uniform gratings. Additionally, only the main peak is considered in the fit by applying a threshold of 20% from the peak value to reject the side lobes of the Bragg reflections. The errors from the specified Bragg wavelengths are determined, and the means and standard deviations for the individual gratings are calculated. In Figure 6b, it can be seen that the Bragg wavelengths can be determined with almost zero bias and a standard deviation of 0.9 pm on average, if the TMM uses the positions estimated in the previous step. This meets the requirements of most applications, usually tolerating errors of up to 1 pm [39].

In order to examine the influence of the position estimation errors, the reflectivity estimation with the TMM is repeated, using the true positions as an input. In this case, the standard deviation reduces to 0.3 pm on average. This indicates that further improvement of the position estimation algorithm could also improve the TMM estimation and Bragg wavelength precision, e.g., by compensating the static offsets observed in Figure 5b or implementing a more advanced probabilistic model for the EDA [38]. However, as the observed errors are already at a very low level with less than 1 pm, the improvement may not be significant in a real measurement scenario. There, additional effects not covered in our simulation like polarization dependencies or the inaccuracy of the laser wavelength might dominate the uncertainty already.

## 3. Experimental Results

In order to experimentally validate the proposed model-based processing, a measurement series was taken out with the setup depicted in Figure 1. All relevant measurement parameters are listed in Table 2 and define a similar configuration to the previous Monte Carlo simulation. Two FBG arrays, each containing 10 identical gratings, were connected together. The gratings were fabricated in a boron co-doped photosensitive single mode fiber (SMF), using a 248 nm UV laser and a phase mask. The reflectivities of the gratings ranged from 0.3–0.8% and the nominal Bragg wavelengths of the gratings were 1540 nm at a temperature of 20 ${}^{{}^{\circ}}$C. The distance between the FBGs was 20 cm and 30 cm in the first and second array, respectively.

The modulation frequency $f_{k}$ was stepped from 10 MHz–500 MHz in steps of Δf=10MHz. This was the same grid as chosen in the simulation, resulting in N=50 measurement frequencies. The wavelength is sequentially tuned over a range of 2 nm in a 40 pm grid, resulting in 51 measurement wavelengths.

Using an intermediate frequency bandwidth of $B_{\mathrm{IF}}=1\;\mathrm{kHz}$, a single frequency sweep of the VNA took about 43 ms, which added up to 2.2 s for all measurement wavelengths $\lambda_{n}$. One problem however was that the TLS required some time for wavelength and power stabilization, which added another 0.25 s dead time to each VNA sweep. This increased the measurement time for a complete interrogation to 15 s in total.

By using the model-based approach, no IDFT with its limitations and compromises had to be performed. Instead, the positions and the reflectivities were directly estimated from the measured frequency domain data $H(f_{k},\lambda_{n})$. This was done by estimating the positions of the gratings in a first step, using Algorithm 1 as outlined in Section 2.2.2. In a second step, the resulting estimated positions $z_{m}^{*}$ were used as static parameters in the TMM and the reflectivities $R_{m}\left(\lambda_{n}\right)$ were estimated with a least squares fit for each wavelength as described in Section 2.2.3. In contrast to the simulations, the true parameters of the FBGs were not exactly known in the measurements. While this makes an evaluation of systematic errors unfeasible, we do not consider this as very critical, as the simulation results under similar conditions showed negligible systematic position errors in Figure 5b and no offsets for the Bragg wavelengths in Figure 6b. Consequently, it is sufficient to only evaluate the precision of the estimation by analyzing the variance of several repeated measurements.

**Figure 7:**
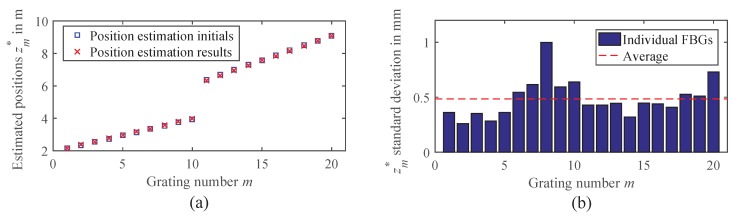
(**a**) Example result of the position estimation algorithm for a measurement taken at 25 ${}^{{}^{\circ}}$C and $B_{\mathrm{IF}}=1\;\mathrm{kHz}$. (**b**) Standard deviation of the estimated positions, resulting from 100 repeated measurements at 25 ${}^{{}^{\circ}}$C.

The FBG arrays were placed inside of a climate chamber (CTS, C-40/100), which controlled the temperature from −20 ${}^{{}^{\circ}}$C–85 ${}^{{}^{\circ}}$C in steps of 15 K. A series of 100 measurements was taken for each temperature to collect a reasonable amount of data for a statistical analysis of the Bragg wavelength errors. Figure 7a shows an example of the position estimation for a measurement taken at 25 ${}^{{}^{\circ}}$C. The initial positions for the estimation are also depicted, which were chosen in the nominal grid of 20 cm and 30 cm. The estimated positions revealed some slight variations from the nominal grid due to manufacturing tolerances, which could be handled well by the position estimation algorithm. Analyzing the standard deviation of the estimated positions, an average precision of 0.5 mm was achieved, as depicted in Figure 7b.

**Figure 8:**
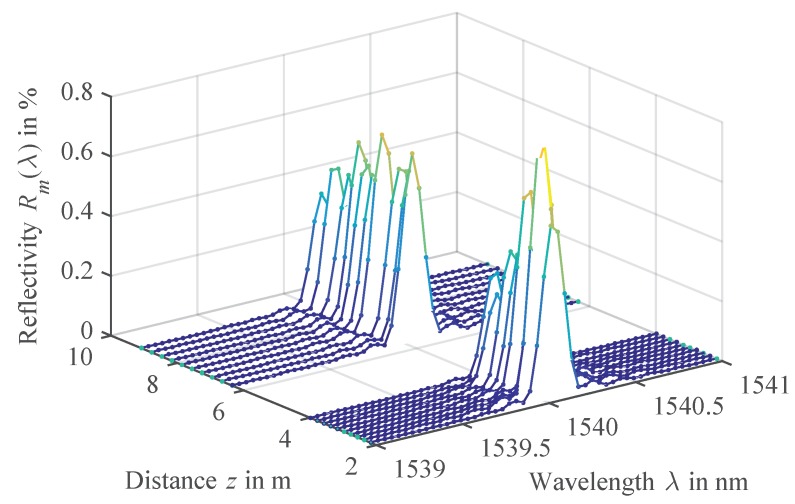
Example result of the FBG reflectivities $R_{m}\left(\lambda_{n}\right)$ estimated from a measurement at 25 ${}^{{}^{\circ}}$C and $B_{\mathrm{IF}}=1\;\mathrm{kHz}$ using the proposed EDA position and transfer matrix model (TMM) reflectivity estimation algorithms.

After the positions have been estimated, the reflectivities are estimated with the TMM. Figure 8 shows an example result of the reflectivity estimation. The FBG reflection profiles are reconstructed well by the TMM estimation and even the weak side lobes of the gratings can be resolved clearly. Again, the Bragg wavelengths $\lambda_{B,m}$ are extracted from this by fitting a Gaussian profile to the main peaks.

**Figure 9:**
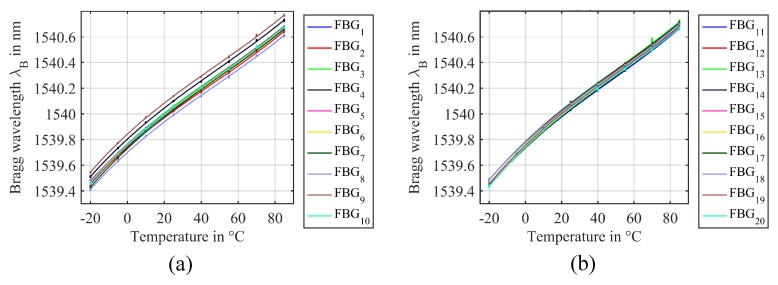
Evaluated Bragg wavelengths $\lambda_{B}$ in the temperature range from −20 ${}^{{}^{\circ}}$C–85 ${}^{{}^{\circ}}$C and third order polynomial curve fits. Dots represent the Bragg wavelengths of 100 repeated measurements for each temperature. (**a**) First ten gratings. (**b**) Last ten gratings.

We also recorded the temperature characteristics of the Bragg wavelengths, which are depicted in Figure 9. In the temperature range from 10 ${}^{{}^{\circ}}$C–85 ${}^{{}^{\circ}}$C, linear temperature sensitivities of 10.5 pm/K can be observed, which is a common value for FBGs in a boron co-doped photosensitive fiber [7]. At lower temperatures, however, some nonlinearity of the temperature sensitivity could be observed. We suspect this to be an influence of the re-coating material that was used for the FBGs after manufacturing, causing a slight compression of the FBGs at temperatures below 0 ${}^{{}^{\circ}}$C. Such a nonlinear characteristic can be handled by fitting a higher polynomial to the temperature curves. In our experiment, we found a third order polynomial to fit the temperature curves in Figure 9 sufficiently well, for example. Besides such nonlinearities, one would also have to take the packaging and the attachment of the sensors to a measurement object into account, which is however beyond the scope of this paper.

**Figure 10:**
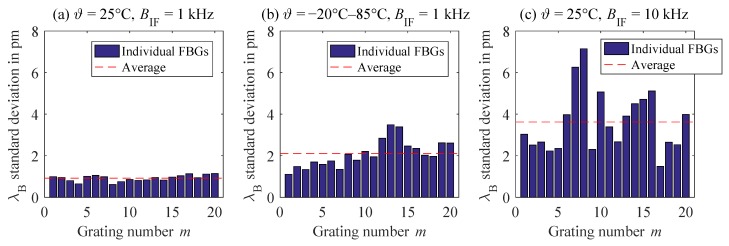
Evaluation of the standard deviation of the Bragg wavelengths obtained from 100 repeated measurements for each temperature. (**a**) Results for 25 ${}^{{}^{\circ}}$C and $B_{\mathrm{IF}}=1\;\mathrm{kHz}$. (**b**) Results for all temperatures from $-20{\;}^{{}^{\circ}}C$–$85{\;}^{{}^{\circ}}C$ in 15 K steps and $B_{\mathrm{IF}}=1\;\mathrm{kHz}$. (**c**) Results for 25 ${}^{{}^{\circ}}$C and $B_{\mathrm{IF}}=\;10\;\mathrm{kHz}$.

The variation of the Bragg wavelengths over 100 repeated measurements is shown in Figure 10a, measured at 25 ${}^{{}^{\circ}}$C and an IF bandwidth of $B_{\mathrm{IF}}=1\;\mathrm{kHz}$. On average, a low standard deviation of 1 pm was observed, which agreed with the simulation results reasonably well. Considering the temperature sensitivity observed in Figure 9, this would correspond to a temperature repeatability of about 0.1 K. Taking all temperature measurements between $-20^{\;{}^{\circ}}C$ and $85\;{}^{{}^{\circ}}C$ into account, the average standard deviation increased to 2 pm, as shown in Figure 10b. This increase was mainly caused by temperature variations of the climate chamber controlling higher and lower temperatures, but was still well inside its temperature stability specification of ≤0.3 K. We also examined the penalty of increasing the intermediate bandwidth to 10 kHz. This reduced the sweep time of the VNA to 4.3 ms per measurement wavelength, but also decreased the SNR by approximately a factor of ten. In this case, an increase of almost 3 pm of the standard deviation at 25${}^{{}^{\circ}}$ is observed in Figure 10c.

## 4. Discussion

Using the proposed model-based estimation methods, a very efficient signal processing of FBG arrays can be performed. In summary, the positions of twenty gratings in spacings of 20 cm and 30 cm could be estimated with a precision of 0.5mm, and the Bragg wavelengths could be determined with 2pm repeatability in the temperature range from $-20{\;}^{{}^{\circ}}C$–$85{\;}^{{}^{\circ}}C$, corresponding to a precision of about 0.2K. In previously performed work [7], a comparable standard deviation of less than 0.3K was achieved, however using a much larger measurement bandwidth of B=4.8GHz and N = 800 measurement frequencies. Using model-based processing, we achieved a slightly better precision, using an electrical bandwidth of only 500 MHz and N = 50 measurement frequencies, providing a significant saving of electrical bandwidth and measurement time without penalties in precision. Besides this, the model-based processing of the iOFDR measurements yields a number of other advantages compared to the conventional IDFT processing:

- An FBG array can be modeled using a sparse set of parameters (e.g., reflectivities and positions), so only a small number of parameters needs to be estimated. Priorly known or estimated parameters (e.g., grating positions) can be included in the models so that the number of estimation parameters reduces even further, saving computational time.
- The drawbacks of IDFT processing associated with windowing can be avoided as the parameters are directly estimated in the frequency domain.
- The modulation frequencies $f_{k}$ are not bound to an equidistant grid as required for IDFT processing, but could be chosen irregularly or randomly, instead.
- Systematic errors due to crosstalk by multiple reflections and spectral shadowing between gratings at the same wavelength can be mitigated using the TMM, as it considers the forward and backward propagation of the modulation signal.

The last point in the list could be especially relevant for large FBG arrays with many gratings at the same wavelength, as crosstalk errors increase with the number of gratings and their reflectivity [21,25]. Using a combination of WDM/TDM for example, very large sensor networks containing up to thousands of FBG sensors could be potentially realized; however, these would also suffer from increased crosstalk errors. So far, this crosstalk could only be suppressed by using ultra-weak gratings, decreasing the reflected signals to very low levels [22,23]. In order to test the suitability of our model-based processing also for such large-scale FBG arrays, we perform a further simulation in the following:

We simulate an array containing M=200 equal FBGs in a grid of ΔL=25cm, resulting in a fiber length of 50 m. Similar to the simulations in Section 2.3, we slightly randomize the grating positions $z_{m}$, Bragg wavelengths $\lambda_{B,m}$, peak reflectivities $R_{B,m}$ and spectral bandwidths $\Delta \lambda_{\mathrm{FWHM}}$ to consider manufacturing tolerances and temperature variations. As the array contains ten times more gratings than before, we reduce the nominal peak reflectivities of the gratings to $R_{B,m}=0.05\%$ to keep the amount of crosstalk at a similar level as in the simulations and measurements regarded before. Due to the increased fiber length, the modulation frequency step also needs to be reduced according to Equation (2). We choose an equidistant grid with steps of Δf=1MHz, while keeping an electrical bandwidth of B=500MHz, resulting in N=500 measurement frequencies. All other parameters and the RMS value of the additional noise $\sigma_{n}$ are kept the same as listed in Table 1.

**Figure 11:**
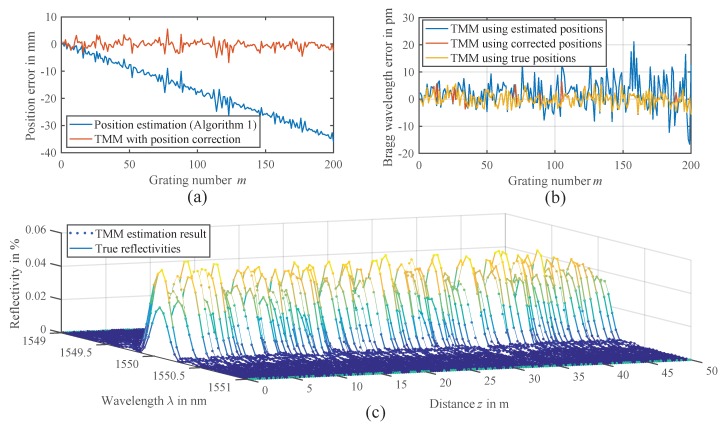
Model-based estimation results of a simulated FBG array with M=200 gratings in a nominal grid of ΔL=25cm. (**a**) Position estimation error of Algorithm 1 and position correction. (**b**) Bragg wavelength errors when using the estimated, corrected or true positions in the TMM estimation. (**c**) Comparison of true (solid lines) and estimated reflectivity profiles (dots).

The estimation results are depicted in Figure 11. As before, we first estimate the grating positions $z_{m}$, using the EDA-based Algorithm 1 with a nominal grid of 25 cm as the initial positions. The resulting estimated positions are plotted in Figure 11a. As already observed in Figure 5b, to some extent, the estimated positions show a systematic position error. Unfortunately, this error adds up to 35 mm for the last FBGs in the given array and also influences the following estimation of the Bragg wavelengths with the TMM. As a result, an increased average Bragg wavelength error of 5.5 pm is observed in Figure 5b, compared to 2.4 pm, which would have been achieved if the true positions had been known.

We suspect that the systematic position estimation error occurs due to the simplifications made in the position estimation model (14), only considering the direct reflections of the gratings and neglecting any crosstalk. This was not an issue for smaller arrays, examined in the simulations and experiments before, but becomes significant for a large number of FBGs. One solution could be a modification of the model to compensate for this systematic error. We however choose a different approach and include an estimation of this residual position error in the TMM estimation. This can be achieved by simply substituting the lengths of the FBG spans $L_{m}$ by the estimated span lengths minus the estimation errors $L_{m}$ = ${\tilde{L}}_{m}-\delta L_{m}$. By also performing an estimation of $\delta L_{m}$ with the least squares fitting routine parallel to the reflectivities, the systematic position error can be completely compensated.

Figure 11a shows the remaining position errors corrected by the estimated systematic position errors $\delta L_{m}$, leaving only a stochastic variation with a standard deviation of 1.6 mm. This significantly improves the TMM performance estimating the Bragg wavelengths, so that a standard deviation of the Bragg wavelength error can be reached that is just 0.1 pm worse than obtained from the estimation using the true positions. Figure 11c shows the resulting reflectivity profiles $R_{m}\left(\lambda \right)$ of the 200 FBGs using the position correction, compared to the true reflectivities as specified in the simulation.

It can be clearly seen that the gratings can be reconstructed very well without any deformations due to multiple reflections or power depletion caused by spectral shadowing [21,25]. The results from Figure 11c indicate that the proposed model-based processing could be used to compensate such crosstalk, as the TMM considers spectral shadowing by the multiplication of subsequent FBG transfer matrices and also the back and forth propagation of multiple reflections. Therefore, we see a potential that further improvements of large-scale FBG array sensing could be achieved with the developed model-based processing.

As a further outlook, other FBG measurement techniques, in particular coherent OFDR systems [1,8], could potentially benefit from the proposed model-based approach, as well. There, the reflection profile of an FBG array R(z) is also obtained by a Fourier transform of an interferogram, which is very similar to iOFDR processing. This Fourier transform processing could be replaced by model-based estimations, using similar transfer matrices to model the interferometric response of an FBG array for these systems.

Of course, some limitations also exist for model-based processing that are subject to further optimization; most notably, an increased computational effort and the complexity of the models. For example, the simplified frequency response model (13) in the EDA position estimation algorithm reduced the estimation problem to a simple linear equation system, saving computational time, but also caused some errors in the position estimation results. Therefore, there will be always a trade off between accuracy and processing speed, which needs to be optimized for every application.

The computational effort also strongly depends on the prior knowledge of a given sensor array. For example, if the number of gratings were not known in advance, additional classification algorithms would be required to determine the model dimension *M* before the position and reflectivity estimation could be performed. On the other hand, effort could also be reduced for parameters that are not subject to large fluctuations. For example, the FBG positions experience only small changes of the optical path length due to temperature variations, which could be compensated by the position error correction method that is suggested in this section. Consequently, the estimation of the grating positions with the EDA could be performed more occasionally than the reflectivity estimation, saving computational time.

Another aspect to note is that model-based processing is most efficient if the system can be expressed by a sparse parametric model. In the case of quasi-distributed FBG sensing with the iOFDR technique for example, an FBG can be modeled using just two parameters, namely its position and reflectivity at a given wavelength. This sparse modeling removes much redundancy from the processing and is the main reason for the improvements of the model-based method compared to the conventional Fourier transform processing, which would require larger bandwidth and more measurement frequencies to achieve comparable results.

## 5. Conclusions

In this paper, we gave a detailed introduction to a model-based processing for the position and reflectivity estimation of quasi-distributed FBG arrays with the iOFDR technique. The model-based processing concept provides much flexibility, as models can be specifically tailored to the sensor configuration and prior knowledge of the sensors (e.g., positions) can be considered in the estimations. Monte Carlo simulations and measurements demonstrated that the proposed model-based approach outperforms conventional processing techniques based on a Fourier transform, especially near the spatial resolution limit. In the experiments, we could achieve a temperature precision of about 0.2 K, using only a tenth of the electrical bandwidth and measurement frequencies that would have been required for conventional Fourier transform processing to achieve a similar precision. This significantly saves system costs and measurement time and provides a much more efficient processing. The estimation algorithms also performed well in simulations for large-scale arrays, which could be beneficial for future large TDM/WDM sensor networks. There, the developed TMM could be especially useful to compensate crosstalk errors due to multiple reflections and spectral shadowing, as the model considers the forward and backward propagation of the modulation signal.

In conclusion, we could demonstrate that model-based processing can be a very efficient and advantageous processing technique in optical fiber sensing, further improving system performance. 

## Figures and Tables

**Table 1:** Parameters as specified for the simulated frequency responses $H(f_{k},\lambda_{n})$.

Parameter	Symbol	Value ± Tolerance ^a^	Unit
Number of FBGs	*M*	20	–
Bragg wavelengths	$\lambda_{B,m}\pm \sigma_{\lambda_{B}}$	1550 ± 0.1	nm
FBG spectral bandwidth (FWHM)	$\Delta \lambda_{\mathrm{FWHM}}\pm \sigma_{\Delta \lambda }$	200 ± 20	pm
FBG peak reflectivities	$R_{B,m}\pm \sigma_{R_{B}}$	0.5 ± 0.1	%
FBG positions m=1,2,…,10	$z_{m}\pm \sigma_{z}$	2.0, 2.2, …, 3.8 ± 0.02	m
FBG positions m=11,12,…,20		5.8, 6.1, …, 8.5 ± 0.03	
Number of simulation runs	$N_{\mathrm{sim}}$	1000	–
Modulation frequencies	$f_{k}$	10 …500	MHz
Modulation frequency steps	Δf	10	MHz
Measurement wavelengths	$\lambda_{n}$	1549…1551	nm
Measurement wavelength steps	Δλ	40	pm
Additional Gaussian noise (RMS)	$\sigma_{n}$	$1.5\times 10^{-5}$	–
Fiber group refractive index	$n_{g}$	1.447	–
Fiber attenuation coefficient	α	0.25	dB/km

^a^ Tolerance values specify the standard deviation *σ* of a Gaussian distribution.

**Table 2:** Measurement parameters.

Parameter	Symbol	Values	Unit
Measurement temperature range	*T*	−20…85	${}^{{}^{\circ}}$C
Measurement temperature steps	ΔT	15	K
Number of measurements per temperature	$N_{\mathrm{meas}}$	100	–
Modulation frequencies	$f_{k}$	10 …500	MHz
Modulation frequency steps	Δf	10	MHz
VNA intermediate frequency bandwidth	$B_{\mathrm{IF}}$	1	kHz
Measurement wavelengths	$\lambda_{n}$	1539…1541	nm
Measurement wavelength steps	Δλ	40	pm
Average fiber coupled optical power	$P_{0}$	−5	dBm
Fiber group refractive index	$n_{g}$	1.447	–
Fiber attenuation coefficient	α	0.25	dB/km

## Appendix A

The least squares problem (12) can be solved in a closed form for the reflectivities ${\bar{R}}_{m,i}^{*}$, given the positions $z_{m,i}^{*}$ (m=1,2,…,M). Rewriting the equation system of the simplified model (14) for $\bar{H}\left(f_{k}\right)$ (k=1,2,…,N) in matrix form gives:

(A1) $$\left(\begin{array}{c} \bar{H}\left(f_{1}\right)\\ \bar{H}\left(f_{2}\right)\\ \vdots \\ \bar{H}\left(f_{N}\right) \end{array}\right)\approx \left(\begin{array}{cccc} e^{-j\frac{4\pi f_{1}}{v_{g}}z_{1,i}^{*}} & e^{-j\frac{4\pi f_{1}}{v_{g}}z_{2,i}^{*}} & \cdots & e^{-j\frac{4\pi f_{1}}{v_{g}}z_{M,i}^{*}}\\ e^{-j\frac{4\pi f_{2}}{v_{g}}z_{1,i}^{*}} & e^{-j\frac{4\pi f_{2}}{v_{g}}z_{2,i}^{*}} & \cdots & e^{-j\frac{4\pi f_{2}}{v_{g}}z_{M,i}^{*}}\\ \vdots & \ddots & \vdots \\ e^{-j\frac{4\pi f_{N}}{v_{g}}z_{1,i}^{*}} & e^{-j\frac{4\pi f_{N}}{v_{g}}z_{2,i}^{*}} & \cdots & e^{-j\frac{4\pi f_{N}}{v_{g}}z_{M,i}^{*}} \end{array}\right)\cdot \left(\begin{array}{c} {\bar{R}}_{1,i}^{*}\\ {\bar{R}}_{2,i}^{*}\\ \vdots \\ {\bar{R}}_{M,i}^{*} \end{array}\right),$$

or in short-form notation:

(A2) $$\bar{H}=\Phi \cdot R^{*}.$$

The phasor matrix Φ consists only of complex constants, so the least squares solution of (12) for the vector $R^{*}$ is known to be [14] (ch. 3.3.1):

(A3) $$R^{*}={\left(\Phi^{T}\Phi \right)}^{-1}\Phi^{T}\bar{H}.$$

## Appendix B

Partial derivatives of the TMM frequency response (9) with respect to the reflectivity estimation parameters $R_{m}\left(\lambda_{n}\right)$:

(A4) $$\begin{array}{cc} \frac{\partial H_{\mathrm{TMM}}\left(f_{k},\lambda_{n}\right)}{\partial R_{m}\left(\lambda_{n}\right)}= & \left\{\left[{\left(\prod_{\mu =m-1}^{1}T_{\mathrm{Span},\mu }\right)}_{11}+H_{\mathrm{TMM}}\left(f_{k},\lambda_{n}\right){\left(\prod_{\mu =m-1}^{1}T_{\mathrm{Span},\mu }\right)}_{12}\right]e^{-j\phi_{m}\left(f_{k}\right)}\right.\\ & -\left.\left[{\left(\prod_{\mu =m-1}^{1}T_{\mathrm{Span},\mu }\right)}_{21}+H_{\mathrm{TMM}}\left(f_{k},\lambda_{n}\right){\left(\prod_{\mu =m-1}^{1}T_{\mathrm{Span},\mu }\right)}_{22}\right]e^{j\phi_{m}\left(f_{k}\right)}\right\}\\ & \cdot \frac{{\left(\prod_{\mu =M}^{m+1}T_{\mathrm{Span},\mu }\right)}_{21}+{\left(\prod_{\mu =M}^{m+1}T_{\mathrm{Span},\mu }\right)}_{22}}{{\left(1-R_{m}\left(\lambda_{n}\right)\right)}^{2}{\left(\prod_{\mu =M}^{1}T_{\mathrm{Span},\mu }\right)}_{22}}. \end{array}$$
